# Influence of acute and chronic intermittent hypoxic-hyperoxic exposure prior to aerobic exercise on cardiovascular risk factors in geriatric patients—a randomized controlled trial

**DOI:** 10.3389/fphys.2022.1043536

**Published:** 2022-10-28

**Authors:** Tom Behrendt, Ann-Christin Altorjay, Robert Bielitzki, Martin Behrens, Oleg S. Glazachev, Lutz Schega

**Affiliations:** ^1^ Department for Sport Science, Chair for Health and Physical Activity, Otto-von-Guericke University Magdeburg, Magdeburg, Germany; ^2^ Department of Internal Medicine, Division of Cardiology and Angiology, University Hospital Magdeburg, Magdeburg, Germany; ^3^ Department of Orthopedics, University Medicine Rostock, Rostock, Germany; ^4^ Departement Human Physiology, Institute of Clinical Medicine, I.M. Sechenov First Moscow State Medical University, Moscow, Russia

**Keywords:** hypoxia-hyperoxia, training, lipoprotein, cholesterol, elderly, aging

## Abstract

**Background:** Intermittent hypoxic-hyperoxic exposure (IHHE) and aerobic training have been proposed as non-pharmacological interventions to reduce age-related risk factors. However, no study has yet examined the effects of IHHE before aerobic exercise on cardiovascular risk factors in the elderly. Therefore, the aim of this study was to investigate the acute and chronic effects of IHHE prior to aerobic cycling exercise on blood lipid and lipoprotein concentrations as well as blood pressure in geriatric patients.

**Methods:** In a randomized, controlled, and single-blinded trial, thirty geriatric patients (72–94 years) were assigned to two groups: intervention (IG; *n* = 16) and sham control group (CG; *n* = 14). Both groups completed 6 weeks of aerobic cycling training, 3 times a week for 20 min per day. The IG and CG were additionally exposed to IHHE or sham IHHE (i.e., normoxia) for 30 min prior to aerobic cycling. Blood samples were taken on three occasions: immediately before the first, ∼10 min after the first, and immediately before the last session. Blood samples were analyzed for total (tCh), high-density (HDL-C), and low-density lipoprotein cholesterol (LDL-C), and triglyceride (Tgl) serum concentration. Resting systolic (SBP) and diastolic blood pressure (DBP) was assessed within 1 week before, during (i.e., at week two and four), and after the interventions.

**Results:** The baseline-adjusted ANCOVA revealed a higher LDL-C concentration in the IG compared to the CG after the first intervention session (η_p_^2^ = 0.12). For tCh, HDL-C, Tgl, and tCh/HDL-C ratio there were no differences in acute changes between the IG and the CG (η_p_^2^ ≤ 0.01). With regard to the chronic effects on lipids and lipoproteins, data analysis indicated no differences between groups (η_p_^2^ ≤ 0.03). The repeated measures ANOVA revealed an interaction effect for SBP (η_p_^2^ = 0.06) but not for DBP (η_p_^2^ ≤ 0.01). Within-group post-hoc analysis for the IG indicated a reduction in SBP at post-test (d = 0.05).

**Conclusion:** Applying IHHE prior to aerobic cycling seems to be effective to reduce SBP in geriatric patients after 6 weeks of training. The present study suggests that IHHE prior to aerobic cycling can influence the acute exercise-related responses in LDL-C concentration but did not induce chronic changes in basal lipid or lipoprotein concentrations.

## 1 Background

Aging impairs the state and function of almost all major organ systems including the nervous (Kalpouzos et al., 2012; Hoogendam et al., 2014), cardiovascular (Gude et al., 2018; Ungvari et al., 2018), and neuromuscular system (Hairi et al., 2010; Keller and Engelhardt, 2013; Mau-Moeller et al., 2013; Reuter et al., 2015). These alterations increase the risk of morbidity and mortality. Geriatric medicine is concerned with the structural, functional, and molecular changes as well as the diseases in elderly in order to prescribe medical care and treatment. In general, geriatric patients can be described as a subgroup of frail older people suffering from multiple comorbidities that usually have significant functional implications (Hilmer et al., 2007). In this regard, a cross-sectional study screening 3292 geriatric patients older than 65 years found that hypertension and dyslipidemia were the most common chronic comorbidities in this cohort (Barrio-Cortes et al., 2021).

High blood pressure is the leading risk factor for cardiovascular disease mortality and disease burden (The Global Burden of Metabolic Risk Factors for Chronic Diseases Collaboration, 2014; GBD 2017 Risk Factor Collaborators, 2018). Although the regulation of blood pressure is determined by a complex interaction of multiple organs and physiological mechanisms, cardiac output and systemic vascular resistance are thought to be the most important factors (Lionakis et al., 2012; Alshami et al., 2018). Among others, impaired endothelium-dependent nitric oxide (NO)-mediated vasodilatation (i.e., endothelial dysfunction) is one of the leading mechanism for increased vascular resistance and thus hypertension (Alvis and Hughes, 2015). During the aging process, an increased expression of vasoconstrictor factors and pro-inflammatory cytokines, oxidative stress, and cell senescence as well as a decrease in NO bioavailability and production contribute to endothelial dysfunction (Herrera et al., 2010; Ungvari et al., 2018). Furthermore, the current evidence indicate that older people have an increased sympathetic activity (Seals and Esler, 2000) likely due to, among others, a diminished sympathetic baroreflex sensitivity, which may predisposed them to an increased prevalence of hypertension (Okada et al., 2012).

Dyslipidemia is commonly characterized by changed lipid and lipoprotein levels, more particularly by reduced high-density lipoprotein cholesterol (HDL-C) and/or increased total cholesterol (tCh), low-density lipoprotein cholesterol (LDL-C) and/or triglyceride (Tgl) levels. Studies have shown that HDL-C levels decrease during the aging process, whereas tCh, LDL-C, and Tgl levels increase (Ericsson et al., 1991; Stevenson et al., 1993). In addition, reduced HDL-C levels and increased LDL-C and Tgl levels provide a predictive measure of cardiovascular disease, such as coronary heart disease and myocardial infarction (Sharrett et al., 2001; Hedayatnia et al., 2020). In this regard, a previous meta-analysis showed that lowering LDL-C concentration by about 1 mmol/L with pharmacological treatments (i.e., standard statin regimes) reduced the 5-year incidence of major coronary events, revascularisations, and ischaemic strokes by ∼20% (Cholesterol Treatment Trialsists' Collaboration, 2010). Despite these encouraging effects of pharmacological treatments, it is important to consider that geriatric patients have multiple chronic concomitant diseases (i.e., multiple comorbidities or multimorbidity), which is associated with polypharmacy (commonly defined as the daily intake of five or more medications by one person (Masnoon et al., 2017)). For instance, Barrio-Cortes et al. showed that the prevalence of polypharmacy was 9% in geriatric patients aged 65–75 years, whereas the prevalence increased up to 75% at older ages of 76–85 years (Barrio-Cortes et al., 2021). In this context, polypharmacy is associated with an increased risk of adverse effects due to drug-drug interactions, which are often complex and elusive (Pazan and Wehling, 2021). Accordingly, there is growing interest and urgent need for innovative non-pharmacological intervention strategies that are able to effectively prevent and treat common age-related risk factors and disease, respectively, in older, frail, comorbid individuals, such as geriatric patients.

Generally described as polypill (Pareja-Galeano et al., 2015), physical training (e.g., aerobic training) has been recognized as a valuable intervention strategy for health promotion and disease prevention and/or treatment in older adults. Particularly for aerobic training, these recommendations proposed a duration of 20–60 min per session, a frequency of 3–7 days per week, and an intensity corresponding to 40–70% of the individual’s heart rate reserve (Izquierdo et al., 2021). Studies suggested a greater effect of aerobic training at higher intensities (i.e., ≥ 90% peak heart rate or peak oxygen consumption) to reduce cardiovascular risk factors (e.g., hypertension (Cornelissen and Smart, 2013; Pescatello et al., 2015)) and to increase cardiorespiratory fitness (e.g., peak oxygen consumption (Robinson et al., 2017; Hannan et al., 2018)). However, because high intensity exercises are associated with increased mechanical stress on the locomotor system, it might be challenging or even impossible for geriatric patients to achieve higher exercise intensities without substantial risk due to various physical limitations (Pramsohler et al., 2017; Izquierdo et al., 2021).

In this context, studies have shown that exposure to intermittent normobaric hypoxia can increase the effectiveness of physical training (Schega et al., 2013; Hayes et al., 2014; Muangritdech et al., 2020) and thereby reducing age-related risk factors and the development of diseases (Verges et al., 2015; Lizamore and Hamlin, 2017) without correspondingly elevating the mechanical stress on the musculoskeletal system. Intermittent hypoxic exposure describes a non-invasive and non-pharmacological method that refers to the repeated resting exposure to brief periods of hypoxia interspersed with normoxic periods (Serebrovskaya and Xi, 2016). It is known that intermittent exposure to hypoxia and normoxia is secondary responsible for the expression of several proteins including vascular endothelial growth factor (VEGF) (Forsythe et al., 1996), NO synthase (NOs) (Hu et al., 2002; Muangritdech et al., 2020), and peroxisome proliferator-activated receptor gamma coactivator-1α (PGC-1α) (Zoll et al., 2006). These proteins are involved in activating angiogenesis (Wahl et al., 2013), improving vascular endothelial function (i.e., vasodilation (Vedam et al., 2009)), and regulating mitochondrial biogenesis as well as muscle fatty acid oxidation (Scarpulla, 2011). In order to enhance the activity of transcription factors (e.g., nuclear factor kappa B, hypoxia induced factor 1α), which augments the expression of signalling proteins (e.g., VEGF, NOs, and PGC-1α) and thus the beneficial adaptive effects of the intermittent hypoxic stimulus, it is hypothesized that normoxic periods should be replaced by moderate hyperoxia (fraction of inspired oxygen [F_i_O_2_] = 0.30–0.40, termed intermittent hypoxic-hyperoxic exposure [IHHE]) (Sazontova et al., 2012; Mallet et al., 2020; Burtscher et al., 2022). Previous studies have shown that IHHE is safe and well-tolerated in various clinical populations such as patients with coronary artery disease (Glazachev et al., 2017), ischemic heart disease (Tuter et al., 2018), mild cognitive impairment (Serebrovska et al., 2022), prediabetes (Serebrovska et al., 2019), comorbidities (Dudnik et al., 2018), and in geriatric patients (Bayer et al., 2017b; Behrendt et al., 2022a). Hence, intermittent exposure to hypoxia and hyperoxia is expected to have beneficial effects on two of the most common age-related risk factors/comorbidities (i.e., hypertension and dyslipidemia) and is well applicable in geriatric patients. Recently, the effects of IHHE on health-related outcomes have been systematically reviewed, and it has been found that 3–6 weeks of IHHE can reduce cardiovascular risk factors, particularly blood glucose concentration as well as systolic and diastolic blood pressure (Behrendt et al., 2022b). However, the evidence concerning the effects of IHHE on blood lipid and lipoprotein concentrations are inconclusive (Behrendt et al., 2022b).

To date, no study has investigated the influence of IHHE conducted prior to aerobic exercise on major cardiovascular risk factors in geriatric patients. Therefore, the aim of the present study was to examine the acute effect of IHHE prior to aerobic cycling exercise on blood lipid and lipoprotein concentrations (i.e., tCh, HDL-C, LDL-C, Tgl) in geriatric patients. To obtain further insight into the chronic effects of IHHE in combination with aerobic cycling training on age-related cardiovascular risk factors, training induced changes (i.e., after 6 weeks of training) in lipid and lipoprotein concentrations as well as blood pressure (i.e., resting systolic [SBP] and diastolic blood pressure [DBP]) were also investigated in the present study.

## 2 Materials and methods

### 2.1 Study design

This study was a randomized, two-armed, placebo-controlled intervention trial conducted in two inpatient care facilities for geriatric patients in Magdeburg (Saxony-Anhalt, Germany) from April 12 to 17 August 2022. Due to a limited number of training devices and the different locations of the facilities, the study was divided into two blocks of 8 weeks each. The 8-week block in each facility included a 6-week intervention period as well as 1-week pre-diagnostic and 1-week post-diagnostic. Prior to the start of the study, all participants and, if acquired, their legal guardians were informed about the aims and the procedure of the study. Every patient or their legal guardian signed a written consent form. After agreeing to participate in the study, patients were screened regarding the inclusion and exclusion criteria (see Section 2.2). Afterwards, patients’ age, sex, anthropometric data (i.e., body height and weight), resting SBP and DBP, medication history, and previous diagnosed diseases were recorded. In addition, the Mini-Mental State Examination (MMSE) was used to determine patients’ level of cognitive impairment (Folstein et al., 1975). Furthermore, each patient underwent a 10 min lasting hypoxic test to assess individual sensitivity to hypoxia. During this test, patients had to sit on an armchair and continuously inhaled hypoxic air (F_i_O_2_ = 0.12) through a face mask. The hypoxic air mixture was automatically provided by an altitude breathing therapy device (ReOxy, Ai Mediq S.A., Luxemburg), while the patients’ peripheral oxygen saturation (S_p_O_2_) as well as pulse rate were steadily monitored and stored by the device. Furthermore, the patients were supervised by a physician and monitored using a 12-channel electrocardiogram (cardio 100, custo med GmbH, Germany) during the entire duration of the hypoxic test. Based on the temporal parameters recorded during the hypoxic test (i.e., time to reach the target reduction in S_p_O_2_ [i.e., 80%] and the time required for reoxygenation [i.e., baseline S_p_O_2_]), an individually tailored IHHE program (i.e., intensity and frequency of the hypoxic and hyperoxic periods) was suggested by the hypoxic device for each patient. The maximum duration of a single hypoxic period in the IHHE program was limited to 8 min (e.g., if patient’s S_p_O_2_ did not decrease to 80% during the hypoxic test). For the readjustment of the hypoxic dose during the IHHE training, the hypoxic test was repeated after 3 weeks of the intervention (i.e., immediately prior the 10th training session).

**FIGURE 1:**
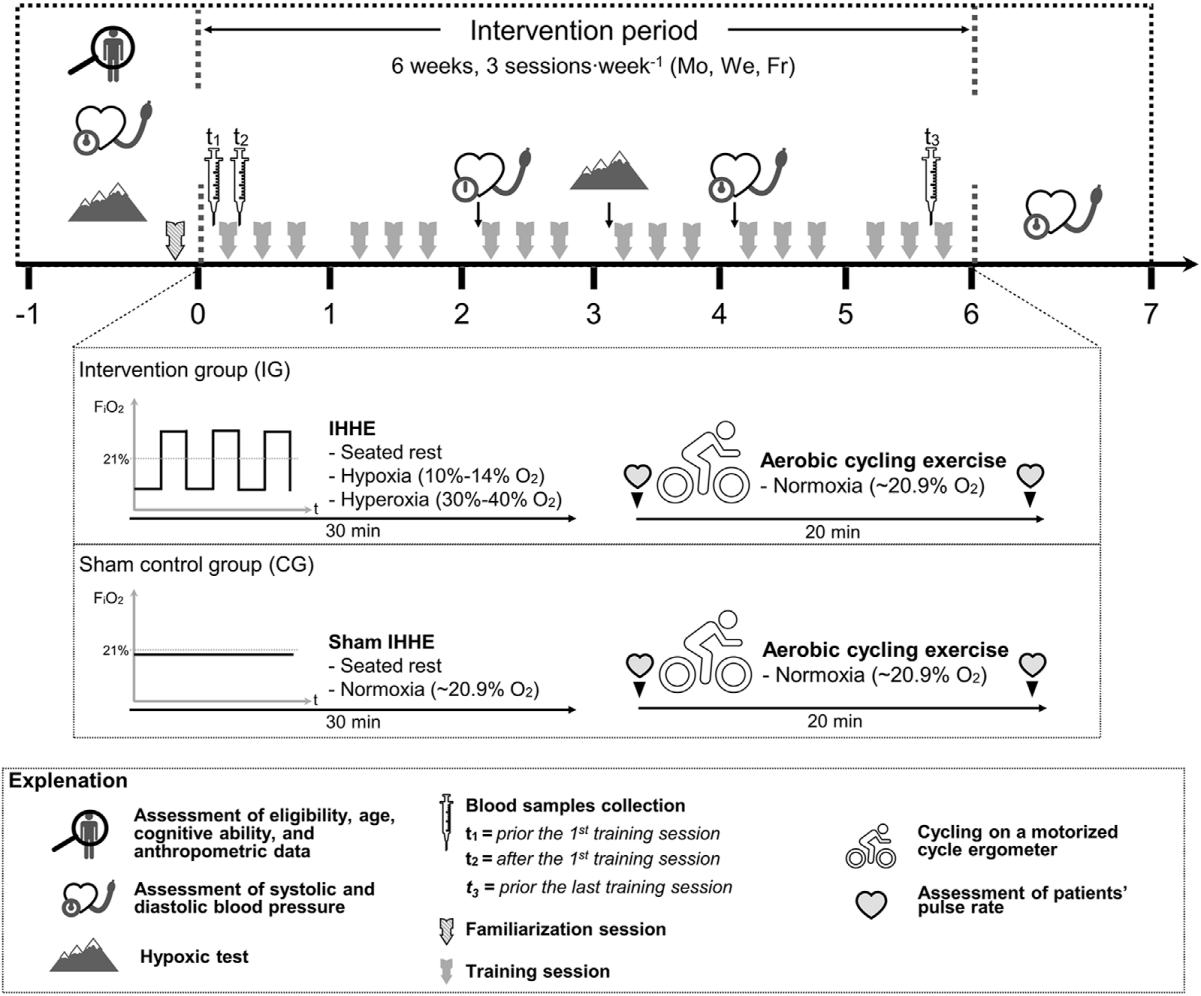
Study design and schematic overview of the interventions. F_i_O_2_, fraction of inspired oxygen; Fr, Friday; IHHE, intermittent hypoxic-hyperoxic exposure; Mo, Monday; We, Wednesday.

Once the pre-diagnostic period was completed, patients were randomly (stratified [MMSE-score] and counterbalanced, allocation ratio 1:1, software: DatInf Randlist v. 1.5, DatInf GmbH Tübingen, Germany) assigned to either the intervention group (IG) or a sham control group (CG). The patients were unaware of their group assignment (single blinded). During the intervention period, blood samples were collected immediately before and ∼10 min after the 1st training session as well as immediately before the last training session (see Section 2.4). Furthermore, patients’ resting SBP and DBP were assessed within 1 week before, after 2 (i.e., prior the 7th training session [2-weeks mid-training]), and 4 weeks (i.e., prior the 13th training session [4-weeks mid-training]), as well as within 1 week after the interventions. Blood pressure was manually measured (inflatable cuff: boso, Germany, 13 × 53 cm cuff size, 12 × 24 cm bladder size; stethoscope: Cordiology IV, 3M, Littmann Stethoscopes, Minnesota, United States) by a trained physician after 10 min of rest using the auscultatory method (Pickering et al., 2005). The arm of the patients was extended and supported by an arm rest at the heart level. An illustration of the study design is shown in Figure 1.

The experimental protocol was approved by the local Ethics Committee of the Otto-von-Guericke University Magdeburg (No. 202/20) confirming to the principles of the Declaration of Helsinki on human experimentation. The study was retrospectively registered at drks. de (DRKS-ID: DRKS00025130).

### 2.2 Participants

**FIGURE 2:**
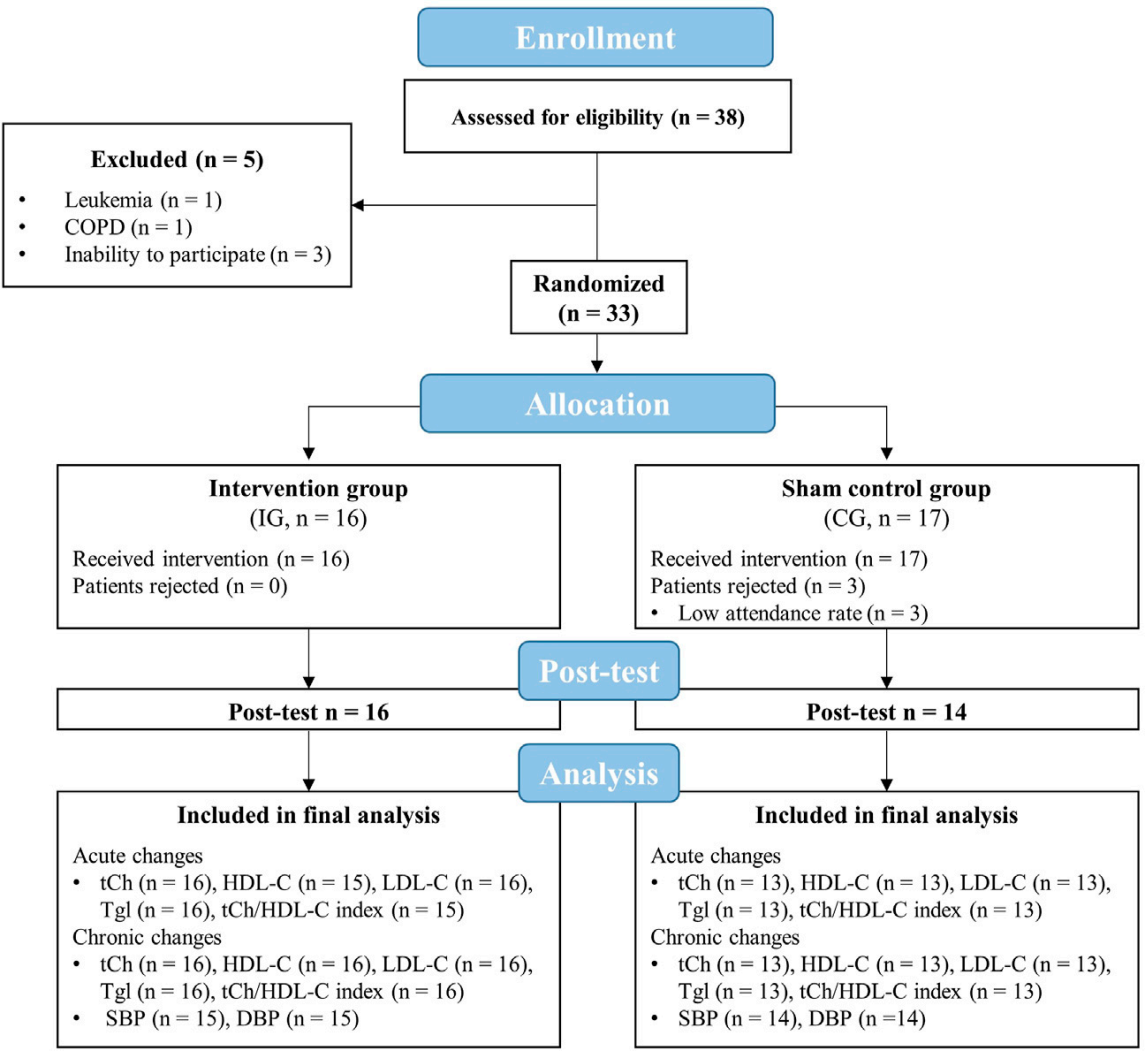
Flow chart of the study. CG, sham control group; COPD, chronic obstructive pulmonary disease; DBP, diastolic blood pressure; HDL-C, high-density lipoprotein cholesterol; IG, intervention group; LDL-C, low-density lipoprotein cholesterol; tCh, total cholesterol; Tgl, triglyceride; SBP, systolic blood pressure.

A total number of 33 geriatric patients were recruited to participate in the present study (IG: *n* = 16, CG: *n* = 17, Figure 2). Patients were part of a larger trial that investigated the effects of an IHHE application prior to aerobic cycling on cognitive and physical performance in geriatric patients (Behrendt et al., 2022a). The sample size for the whole trial was calculated for the primary outcome Dementia-Detection Test performance (Behrendt et al., 2022a) using the software program G*Power (version 3.1), which is not reported in the present study. Based on a medium effect size (f = 0.25), a significance level of 0.05, a power of 0.80, and an expected correlation between measures of 0.7, sample size calculation indicated that a total of 22 patients (11 patients per group) is required to detect potential effects. Patients were included if they were over 60 years of age, non-smoker, have voluntarily agreed to participate, and have given written consent. Exclusion criteria comprised cardiovascular disease (i.e., coronary disease with unstable angina pectoris [CCS 3–4], severe heart failure [NYHA III-IV], arrhythmia), untreated hypertension, untreated or uncontrolled diabetes mellitus, pulmonary fibrosis, cancer, acute inflammatory diseases, need for continuous or intermittent ventilation or oxygenation, resting S_p_O_2_ below 93%, and the simultaneous participation in other interventions.

### 2.3 Intervention

Before the start of the intervention period, patients took part in a familiarization session to get accustomed to the environment and to estimate the patients’ individual level of resistance for the aerobic cycling training. For this purpose, patients were asked to actively pedal on the motorized cycle ergometer starting with the lowest possible resistance level, which was gradually increased afterwards. At each resistance level increment, patients had to indicate whether they thought that the resistance level was appropriate (too easy, too high, and just right (Holthoff et al., 2015)). This approach was used to include patients with obvious cognitive impairment (e.g., dementia). In some patients, neither heart rate nor numerical scales commonly used to assess exercise intensity (e.g., CR-10 scale (Griep et al., 1998) or Borg-scale (Borg, 1982)) could be used. Hence, this alternative method, which was previously used in patients with Alzheimer’s dementia (Holthoff et al., 2015), was chosen.

The training was conducted on 3 days per week (Monday, Wednesday, and Friday) for 6 weeks resulting in 18 training sessions for each patient. The patients in each facility were divided into multiple encounter groups consisting of a maximum of four patients. It was ensured that each encounter group consisted of patients from the IG and CG (ratio 1:1). The training sessions took place in the morning and in the afternoon. Every training session was accompanied by at least one supervisor.

Each training session lasted approximately 60 min and consisted of two parts. In the first part, patients of both groups were connected to an altitude breathing therapy device (ReOxy, Ai Mediq S.A., Luxemburg) through a facemask and inhaled a gas mixture for 30 min while sitting on an armchair. While the IG intermittently inhaled a hypoxic and hyperoxic gas mixture, the CG received a sham mixture with a constant F_i_O_2_ of approximately 0.21 (i.e., normoxia). Afterwards, the face masks were removed and the patients performed 20 min of aerobic cycling exercise using a motorized cycle ergometer (MOTOmed viva two and viva 1, Reck, Germany) under normoxic conditions.

#### 2.3.1 Intermittent hypoxic-hyperoxic exposure

During the IHHE, patients S_p_O_2_ and pulse rate was continuously monitored via a fingertip pulse oximeter (Masimo SET, Switzerland) and stored by the device. When the individual minimum S_p_O_2_ value determined during the hypoxic tests was reached in the course of a hypoxic period, the device automatically switched to hyperoxia until the pre-hypoxia S_p_O_2_ level was achieved. Subsequently, the next hypoxic-hyperoxic cycle started. Depending on the individual tolerance to hypoxia and patients’ acute responses (i.e., S_p_O_2_ and pulse rate), the IG inhaled hypoxia (F_i_O_2_ = 0.10–0.14) for 1–5 min, interspersed by the exposure to hyperoxia (F_i_O_2_ = 0.30–0.40) for 1–3 min. Each program lasted 30 min and consisted of 4–8 hypoxic-hyperoxic cycles. In the course of the IHHE program, the patients from the IG reached an average minimum and maximum S_p_O_2_ of 84 ± 3.4% and 97 ± 1.9%, respectively (Table 1).

**TABLE 1:** Characteristics of the patients at baseline as well as training-related variables.

Characteristics	IG (n = 16)	CG (n = 14)
Age [years]	83.2 ± 4.9	84.3 ± 6.2
Females [n (%)]	15 (94%)	12 (86%)
Height [cm]	158.3 ± 10.6	159.9 ± 8.1
Weight [kg]	71.0 ± 11.2	66.6 ± 10.6
Body Mass Index [cm/kg^2^]	27.8 ± 3.9	26.5 ± 3.4
Mini-Mental-State-Examination	16.8 ± 8.5	14.9 ± 8.0
Clinical Diagnosis [n (%)]		
Alzheimer’s dementia	4 (25%)	7 (50%)
Vascular dementia	1 (6%)	1 (7%)
Mixed types of dementia	6 (38%)	5 (36%)
Hypertension	13 (81%)	12 (86%)
Diabetes mellitus type II	4 (25%)	6 (43%)
Hyperlipidaemia	3 (19%)	3 (21%)
Hypercholesterinaemia	1 (6%)	2 (14%)
Hyperthyreosis	0 (0%)	3 (21%)
Hypothyreosis	2 (13%)	4 (29%)
Regular Medications [n (%)]		
ACE Inhibitors	5 (31%)	3 (21%)
AT1 receptor antagonist	6 (38%)	4 (29%)
β-blockers	7 (44%)	9 (64%)
Calcium channel blockers	4 (25%)	1 (7%)
Diuretics	6 (38%)	3 (21%)
Acetylsalicylic acid	6 (38%)	5 (36%)
Heparinoid	2 (13%)	1 (7%)
Statins	3 (19%)	1 (7%)
Metformin	2 (13%)	1 (7%)
Sulfonylureas	1 (6%)	0 (0%)
Antidiabetics	1 (6%)	3 (21%)
L-Thyroxin	2 (13%)	5 (36%)
Thyreostatics	1 (6%)	2 (14%)
Insulin	1 (6%)	1 (7%)
Variables estimated during the IHHE or sham-IHHE session		
Maximum S_p_O_2_ [%]	97.0 ± 1.9	96.3 ± 1.9*
Minimum S_p_O_2_ [%]	84.1 ± 3.4	94.1 ± 2.6*
Variables estimated before, during, and after the cycling training		
Average work [kJ]	88.8 ± 39.1	101.3 ± 63.7
Heart rate before training [min^−1^]	73.8 ± 9.9	74.6 ± 9.1
Heart rate after training [min^−1^]	75.3 ± 8.9	75.9 ± 10.4

* CG, sham control group; IG, intervention group Effect size d for the between-group differences (d ≥ 0.50).

#### 2.3.2 Aerobic cycling exercise

The aerobic cycling exercise was similar to that used previously (Yang et al., 2014; Holthoff et al., 2015) and included active as well as passive phases. The patients sat on an armchair with their feet fixed to the pedals of the cycle ergometer via Velcro straps. Before the exercise session, pulse rate was measured via a fingertip pulse oximeter (Pulox PO-300, Novidion GmbH, Germany). Thereafter, the patients’ legs were passively moved by the cycle ergometer for 2.5 min at 20 rpm, followed by active pedaling for 15 min at 30–60 rpm. The patients’ pulse rate was again measured after active pedaling via a fingertip pulse oximeter. During the last 2.5 min, the patients performed a passive cool down at 20 rpm. The work (kJ) done by each patient was recorded for every training session. The level of resistance was adjusted by an experienced trainer if necessary.

### 2.4 Blood sample collection and analysis

Blood sample collection was performed at three time points: 1) immediately before the 1st training session (t_1_), 2) ∼10 min after the 1st training session (t_2_), and 3) immediately before the last (18th) training session (t_3_). This approach was utilized to investigate the acute (changes from t_1_ to t_2_) and chronic (changes from t_1_ to t_3_) effects of IHHE or sham-IHHE prior to aerobic cycling exercise on lipid and lipoprotein blood concentrations. In order to keep the conditions as similar as possible for the first (t_1_) and last (t_3_) blood collection (i.e., time of day, day of the week, previous activities throughout the day), the third blood sample was taken immediately before the last (18th) training session.

An 8.5 ml venous blood sample was taken from a superficial forearm vein under stasis conditions from a physician with the patients seated at quiet rest. The blood samples were collected in a vacutainer with separating gel and coagulation activator (BD Vacutainer^®^ II Advance) to determine lipid and lipoprotein serum levels. Immediately after the blood samples were collected, the vacutainer were swirled head down for ten times. Subsequently, the samples were stored for 30 min at room temperature before the serum was separated by centrifugation at 2000 G for 15 min. For each blood collection, 600 μL of serum aliquoted in two tubes were extracted and stored at −80°C for later analysis. Blood serum concentrations of tCh, HDL-C, LDL-C, and Tgl were analyzed by commercial colorimetric kits (Roche Diagnostics, Mannheim, Germany) using Cobas 8000 chemistry analyzer (Roche Diagnostics, Mannheim, Germany).

### 2.5 Statistical analysis

The Shapiro-Wilk test and Levene’s test were used to check the data for normal distribution and homogeneity of variance, respectively. The descriptive statistics were presented as means and standard deviations. Independent sample Student’s *t*-test was used to compare patients’ characteristics at baseline and training variables between the IG and the CG. To check for differences between groups regarding the acute (exercise-induced) and chronic (training-induced) changes in lipid and lipoprotein blood serum concentrations, univariate analyses of covariance (ANCOVA) with baseline values entered as a covariate (baseline-adjusted) were performed. Furthermore, regarding the analysis of acute exercise-induced changes, the average work performed during the aerobic cycling session was entered as a second covariate because studies indicated that the magnitude of acute exercise-induced effects on lipid and lipoprotein concentrations appears to increase with energy expenditure (Thompson et al., 2001). Differences between groups are presented as mean differences together with the 95% confidence intervals to provide information about the magnitude and direction of an effect. Differences in SBP and DBP were tested using 4 (time: pre-training, 2-weeks mid-training, 4-weeks mid-training, post-training) x 2 (group: IG, CG) analyses of variance (ANOVA) with repeated measurements. Furthermore, the classification of blood pressure level (five subdivisions: optimal or normal, high normal, grade 1, 2, and 3 hypertension) at baseline was added as a covariate (Mancia et al., 2013). If the assumption of sphericity was violated, Greenhouse-Geisser correction was applied. In case of significant interaction or main effects, post-hoc tests with Bonferroni correction were performed. Some data did not show variance homogeneity or normal distribution. However, since it was shown that the ANOVA (Schmider et al., 2010; Blanca et al., 2017) and Student’s *t*-Test (Havlicek and Peterson, 1974; Pagano, 2009) are robust to moderate violation of homogeneity and normality assumption, no alternative nonparametric tests were used.

Moreover, the effects sizes partial eta squares (*η*_*p*_^*2*^) and Cohen’s (d) were calculated. A *η*_*p*_^*2*^ between 0.01–0.06, 0.06–0.14 and ≥ 0.14 was considered as a small, medium, and large effect size, respectively (Cohen, 2013; Lakens, 2013). The effect size *d* was used to interpret the results of the Student’s *t*-Tests with 0.20–0.49, 0.50–0.79, and ≥ 0.80 indicating a small, medium, and large effect, respectively (Cohen, 1992). Due to the small sample size, the results of the present study were interpreted based on the effect sizes with a medium effect considered as meaningful (*η*_*p*_^*2*^ ≥ 0.06, *d* ≥ 0.50). Effect sizes are used to determine the practical relevance and generalizability of effects (Lakens, 2013). Data analysis was conducted using JASP Statistics version 0.16 (University of Amsterdam, Amsterdam, Netherlands).

## 3 Results

### 3.1 Patient and training characteristics

Five out of 38 enrolled patients were excluded before randomization due to various reasons (i.e., presence of a disease leading to exclusion or severe cognitive impairment that made participation in the training not possible). Out of these remaining 33 patients that were randomized and assigned to the IG or CG, three had to be excluded from the final analysis due to a low attendance rate (< 80%). Thus, 30 patients (IG = 16, CG = 14) have finished the whole intervention period and had a sufficient training attendance rate (IG = 98 ± 3%, CG = 95 ± 7%). In addition to the eight patients who dropped out, there were irreversible data losses due to measurement errors during the post-test (Figure 2). All components of the intervention (i.e., IHHE and aerobic cycling) were well tolerated by the patients. There were no injuries or adverse side effects except some reports of mild dizziness. Furthermore, some patients mentioned that wearing the face mask was uncomfortable.

The participants’ characteristics (age, height, weight, body mass index), and cognitive ability (operationalized by the MMSE score) of the patients were similar between groups (*p* ≥ .298, *d* ≤ 0.35). The minimum S_p_O_2_ was lower (*T*_*28*_ = 12.322, *p* < .001, *d* = 4.51) and the maximum S_p_O_2_ was higher (*T*_*28*_ = 1.498, *p* = .145, *d* = 0.55) in the IG during IHHE than in the CG during sham IHHE. There were no differences between the IG and CG with respect to the average work (measured in kJ) generated during the 20 min aerobic cycling sessions and the average pulse rate prior as well immediately after these sessions (*p* ≥ .516, *d* ≤ 0.24). Table 1 shows the means (± standard deviations) of the age, the anthropometric and clinical characteristics of the patients as well as the training-related variables.

### 3.2 Blood serum lipid and lipoprotein concentration

At baseline, there were no differences between groups in tCh, HDL-C, and LDL-C (*p* ≥ .235, *d* ≤ 0.45), while Tgl was higher in CG compared to IG (*T*_*28*_ = 1.464, *p* = .155, *d* = 0.55; Table 2).

**TABLE 2:** Acute effects of IHHE (IG) and sham-IHHE (CG) prior to aerobic cycling exercise on lipid and lipoprotein concentrations in blood serum at the first and the last (18th) training session. Data are depicted as pre-exercise and baseline-adjusted post-exercise values (means ± standard deviations) as well as mean differences (MD) (95% confidence intervals [CI]).

Variables [mmol/L]	Group	Pre-training		
		Pre-exercise (t_1_)	Post-exercise (t_2_)	MD_(CG-IG)_ (95% CI) ANCOVA
tCh	IG	5.67 ± 1.28	5.64 ± 0.33	−0.13 (−0.38—0.12)
	CG	5.62 ± 1.46	5.51 ± 0.32	F_1,25_ = 1.188; p = .300; η_p_^2^ = 0.04
HDL-C	IG	1.59 ± 0.36	1.54 ± 0.20	−0.05 (−0.19—0.09)
	CG	1.45 ± 0.43	1.49 ± 0.16	F_1,25_ = 0.631; p = .435; η_p_^2^ = 0.03
LDL-C	IG	3.20 ± 1.07	3.27 ± 0.30	−0.21 (−0.43—0.02)
	CG	3.15 ± 1.25	3.07 ± 0.30	F_1,25_ = 3.385; p = .078; η_p_^2^ = 0.12
Tgl	IG	1.74 ± 0.58	1.97 ± 0.52	−0.05 (−0.47—0.36)
	CG	2.24 ± 1.20	1.91 ± 0.53	F_1,25_ = 0.071; p = .792; η_p_^2^ < 0.01
tCH/HDL-C	IG	3.64 ± 0.88	3.91 ± 0.44	−0.06 (−0.40—0.29)
	CG	4.30 ± 1.94	3.98 ± 0.45	F_1,25_ = 0.113; p = .739; η_p_^2^ < 0.01

CG, control group; HDL-C, high-density lipoprotein cholesterol; IG, intervention group; IHHE, intermittent hypoxic-hyperoxic exposure; LDL-C, low-density lipoprotein cholesterol; tCh, total cholesterol; Tgl, triglyceride.

With regard to the acute effects, the univariate ANCOVA with baseline-adjustment showed differences between groups for acute exercise-related changes in LDL-C (*F*_1,25_ = 3.385; *p* = .078; *η*_*p*_^*2*^ = 0.12). Data analysis revealed a higher LDL-C blood serum concentration with a medium effect size in the IG compared to the CG after an acute exercise session. For tCh, HDL-C, Tgl, and tCh/HDL-C ratio there were no differences in acute exercise-related changes between the IG and the CG. Table 2 shows the lipid and lipoprotein concentrations in blood serum before (t_1,_ pre-exercise) and the baseline-adjusted means and standard deviations after (t_2_, post-exercise) a single exercise session together with the mean differences (95% confidence interval) between the IG and the CG as well as the results of the univariate ANCOVA (*F*-value, df_effect_, df_error_, *p*-value, and effect size *η*_*p*_^*2*^).

With regard to the chronic effects on lipid and lipoprotein blood serum concentrations, there were no differences between groups for tCh, HDL-C, LDL-C, Tgl, and tCh/HDL-C ratio, indicating that the IHHE had no effect on basal lipid or lipoprotein blood serum concentration. Table 3 shows the baseline-adjusted means and standard deviations after the training period (t_3_) as well as the adjusted mean differences (95% confidence intervals) between the IG and the CG together with the results of the univariate ANCOVA (*F*-value, df_effect_, df_error_, *p*-value, and effect size *η*_*p*_^*2*^).

**TABLE 3:** Chronic effects of IHHE (IG) and sham IHHE (CG) prior to aerobic cycling training on lipid and lipoprotein concentrations in blood serum. Data are depicted as baseline-adjusted post-training values as well as mean differences (MD) (95% confidence intervals [CI]).

Variables [mmol/L]	Post-training (t_3_)		MD_(CG-IG)_ (95%CI)	ANCOVA
	IG	CG		
tCh	5.51 ± 0.67	5.40 ± 0.67	-0.10 (-0.62 – 0.41)	F_1,26_ = 0.175; p = .679; η_p_^2^ = 0.01
HDL-C	1.49 ± 0.26	1.54 ± 0.26	0.05 (-0.15 – 0.25)	F_1,26_ = 0.271; p = .607; η_p_^2^ = 0.01
LDL-C	3.29 ± 0.62	3.16 ± 0.62	-0.12 (-0.60 – 0.35)	F_1,26_ = 0.276; p = .604; η_p_^2^ = 0.01
Tgl	1.99 ± 0.60	1.89 ± 0.60	-0.10 (-0.57 – 0.37)	F_1,26_ = 0.188; p = .668; η_p_^2^ = 0.01
tCh/HDL-C	3.89 ± 0.58	3.70 ± 0.58	-0.19 (-0.64 – 0.26)	F_1,26_ = 0.729; p = .401; η_p_^2^ = 0.03

CG, sham control group; HDL-C, high-density lipoprotein cholesterol; IG, intervention group; LDL-C, low-density lipoprotein cholesterol; tCh, total cholesterol; Tgl, triglyceride.

#### 3.3 Systolic and diastolic blood pressure

**FIGURE 3:**
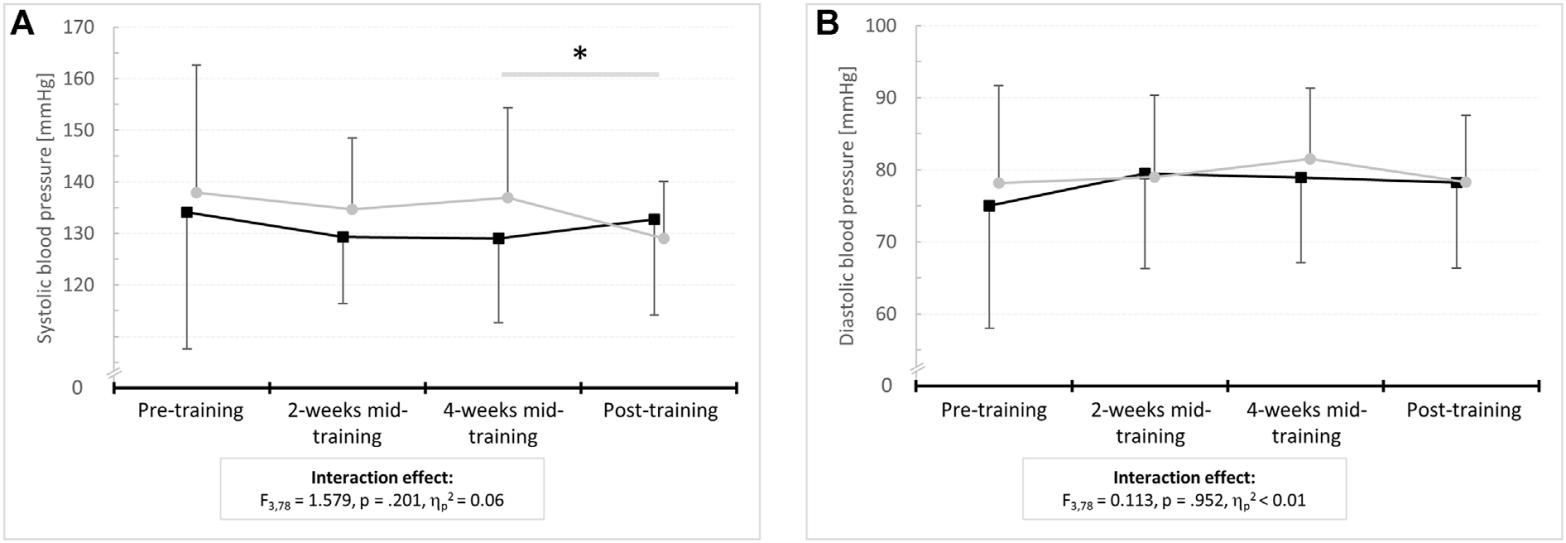
Means and standard deviations for resting **(A)** systolic and **(B)** diastolic blood pressure before (pre-training) as well as after 2 weeks (2-weeks mid-training), 4 weeks (4-weeks mid-training), and 6 weeks of training (post-training) for the intervention group (IG) and sham control group (CG).

There were no differences between the IG and the CG in SBP and DBP (*p* ≥ .586, d ≤ 0.21) at baseline. With regard to SBP, the 4 × 2 ANOVA with repeated measures revealed an interaction effect (*F*_*3*_ *,*_*78*_ = 1.579, *p* = .201, *η*_*p*_^*2*^ = 0.06) as well as a main effect of time (*F*_*3*_ *,*_*78*_ = 8.995, *p* < .001, *η*_*p*_^*2*^ = 0.26) but no main effect of group (*F*_*1*_ *,*_*26*_ = 0.002, *p* = .926, *η*_*p*_^*2*^ ≤ 0.01). Within-group post-hoc analysis for the IG revealed a reduction in SBP by 7.67 mmHg (95% confidence interval: 20.49 to 5.15 mmHg, *p* = .867; *d* = 0.05) at post-test compared to 4-weeks mid-training. No interaction effect or main effect of group (*F*_*1*_ *,*_*26*_ = 0.009, *p* = .926, *η*_*p*_^*2*^ ≤ 0.01) but a main effect of time (*F*_*3*_ *,*_*78*_ = 6.680, *p* < .001, *η*_*p*_^*2*^ = 0.20) was found for DBP. However, post-hoc analysis indicated no within-group differences (*p* = 1.000, d < 0.50). Figure 3 displays the mean values and standard deviations of SBP and DBP for the IG and the CG at pre- and post-test as well as after 2 and 4 weeks of intervention together with the results of the 2 × 4 repeated measures ANOVA (interaction effect: *F*-value, df_effect_, df_error_, *p*-value, and effect size *η*_*p*_^*2*^). Means and standard deviations of these variables as well as the mean differences (95% confidence intervals) between pre- and post-training are also shown in Table 4, together with the statistical parameters for the main effects of group and time (*F*-value, df_effect_, df_error_, *p*-value, and effect size *η*_*p*_^*2*^).

**TABLE 4:** Systolic blood pressure (SBP) and diastolic blood pressure (DBP) prior (pre-training) and 2 (2-weeks mid-training), 4 (4-weeks mid-training) as well as 6 (post-training) weeks after the intervention. Data are depicted as means ± standard deviations as well as mean differences (MD) (95% confidence intervals [CI]).

Variables [mmHg]	Pre-training		2-weeks mid-training		4-weeks mid-training		Post-training		MD_(post-pre)_ (95% CI)		Main effect of group	Main effect of time
	IG	CG	IG	CG	IG	CG	IG	CG	IG	CG		
SBP	137.9 ± 24.7	134.1 ± 26.5	133.8 ± 13.8	129.3 ± 12.8	136.5 ± 16.5	129.0 ± 16.3	128.9 ± 10.7^a^	132.7 ± 18.6	−8.9 (−25.9 – 7.9)	−1.4 (−18.9 – 16.2)	F_3,78_ = 8.995;	F_1,26_ = 0.002;
											p < .001;	p = .962;
											η_p_^2^ = 0.26	η_p_^2^ < 0.01
DBP	78.1 ± 13.5	75.0 ± 17.0	78.1 ± 11.5	79.5 ± 13.2	82.1 ± 9.7	78.9 ± 11.8	78.8 ± 9.0	78.2 ± 11.9	0.2(−12.4 – 12.8)	3.2(−9.9 – 16.3)	F_3,78_ = 6.680;	F_1,24_ = 0.263;
											p = .036;	p = .613;
											η_p_^2^ = 0.17	η_p_^2^ = 0.01

CG, sham control group; IG, intervention group; within-group differences compared to 4-weeks mid-training (d ≥ 0.50).

## 4 Discussion

The present study was designed to determine the acute (i.e., exercise-related) and chronic (i.e., training-related) effects of IHHE prior to aerobic cycling exercise on blood lipid and lipoprotein serum concentrations as well as the resting blood pressure in geriatric patients. The novel finding of this study was that IHHE increased LDL-C concentration in blood serum in response to aerobic cycling exercise. Furthermore, the results demonstrated that an additional IHHE prior to aerobic cycling exercise performed over 6 weeks had no influence on basal lipid and lipoprotein concentrations in blood serum but seemed to be effective to reduce resting SBP.

The present study showed that a single bout of IHHE applied before aerobic cycling increased LDL-C concentration compared to sham IHHE prior to the same exercise. However, no differences were found for the acute changes in tCh, HDL-C, and Tgl concentrations, as well as tCh/HDL-C ratio between the groups. To the best of the authors’ knowledge, there is currently no study available that has examined the acute effects of IHHE prior to aerobic exercise on blood lipid and lipoprotein concentrations. Nevertheless, one previously published study has examined the acute effect of intermittent hypoxia on serum lipids and lipoproteins in humans (Costalat et al., 2018). The results of this study indicated that the relative changes in HDL-C, LDL-C, and Tgl blood concentrations were not different between the hypoxia (i.e., 90 min intermittent hypoxic exposure, F_i_O_2_ = 0.10, average S_p_O_2_ = 80%) and the sham hypoxia (i.e., 90 min normoxia, F_i_O_2_ = 20.9) conditions in overweight/obese subjects. However, the participants did not perform any exercise after the intermittent hypoxia and sham interventions. Studies examining the acute effects of aerobic exercise only on lipid and lipoprotein concentrations showed inconsistent results, reporting both changed (Hicks et al., 1987; Pay et al., 1992; Ferguson et al., 1998; Fittipaldi et al., 2020) and unchanged blood concentrations (Hughes et al., 1991; Imamura et al., 2000; Greene et al., 2012). In particular, studies have found a decrease (Ferguson et al., 1998), no change (Bounds et al., 2000; Antunes et al., 2020), and an increase (Fittipaldi et al., 2020) in LDL-C concentration after acute aerobic exercise. Furthermore, some studies have shown an increase in blood HDL-C (Pay et al., 1992; Fittipaldi et al., 2020) concentrations after prolonged (i.e., 2 h walking) or exhaustive (i.e., maximal exercise test) acute aerobic exercise, while others have reported unchanged concentrations (Hughes et al., 1991; Imamura et al., 2000; Antunes et al., 2020) after low to high intensity continuous aerobic exercise. Considering these conflicting results, it was assumed that the acute effects of exercise (e.g., aerobic exercise) on blood lipid and lipoprotein concentrations are influenced by different factors (Thompson et al., 2001). The key factors include the subjects’ physical fitness level and pre-exercise lipid level as well as the intensity, duration, and mode of exercise (Hicks et al., 1987; Ferguson et al., 1998; Antunes et al., 2020). Although the exact mechanisms remain elusive, it has been suggested that the changes in lipid and lipoprotein concentrations induced by acute aerobic exercise are largely related to increased lipoprotein lipase activity and to a high rate of lipolysis (Pronk, 1993; Thompson et al., 2001). Interestingly, it was shown that lipid oxidation rate (measured *via* indirect calorimetry) was increased 5 min after acute prolonged hypoxic exposure (180 min passive exposure, S_p_O_2_ ∼80%) in sedentary overweight/obese males (Workman and Basset, 2012). Moreover, the acute exposure to continuous hypoxia (6 h, F_i_O_2_ = 0.12) has previously been shown to result in a 95% greater increase in circulating non-esterified fatty acids than under normoxia in fasting healthy males (Mahat et al., 2018), suggesting an increase in adipose tissue lipolysis (Hodson et al., 2008). However, the increase in non-esterified fatty-acids had no effect on fatty acid oxidation throughout the 6 h of hypoxic exposure, as indicated by indirect calorimetry measurements (Mahat et al., 2018). This is in line with the results of a previous study showing that continuous hypoxic training (60 min cycling at 50% of individual peak power output, F_i_O_2_ = 0.15) led to an increased lipid oxidation rate in active healthy males 40 min post-exercise measured under normoxic conditions, whereas it remained unchanged during the exercise session under hypoxia (Kelly and Basset, 2017). Indeed, a review suggests that fatty acid oxidation is attenuated, while glucose uptake is maintained or increased in mammalian skeletal muscle during hypoxic exposure, perhaps to optimize adenosine triphosphate synthesis in the light of a downregulation of the oxidative metabolism (e.g., β-oxidation, oxidative phosphorylation) (Horscroft and Murray, 2014). Noteworthy, studies examining the acute effect of hypoxia on glucose homeostasis point in two directions. On the one hand, studies using intermittent hypoxia protocols with a more severe intensity (F_i_O_2_ = 0.05) and high intra-session frequency (∼25 cycles∙h^−1^) reported reduced insulin sensitivity and increased blood glucose levels in healthy adults (Louis and Punjabi, 2009; Newhouse et al., 2017). Further, animal studies suggest that “high-dose” hypoxia protocols (F_i_O_2_ ≤ 0.07, intra-session frequency ≥ 60 cycles∙h^−1^ (Jun et al., 2014)) stimulate hepatic glycogenolysis, thereby leading to an increase in blood glucose concentration (Rafacho et al., 2013). On the other hand, exposures to intermittent hypoxia protocols with a rather “moderate dose” (F_i_O_2_ = 0.10–0.13, intra-session frequency ∼5–10 cycle∙h^−1^) have been reported to acutely reduce blood glucose concentration in overweight/obese subjects (Costalat et al., 2018) and patients with type II diabetes (Duennwald et al., 2013). This was potentially mediated by, among others, an increased glucose utilization as a result of enhanced activation of hypoxia-induced factor-1α stimulated pathways, adenosine monophosphate-activated protein kinase, and calmodulin-dependent protein kinase (van Hulten et al., 2021). Overall, these results indicate that the acute metabolic response to a hypoxic stimulus depends, among other factors, on several variables that determine the hypoxia dose such as intensity and intra-session frequency. In addition, it appears that acute hypoxia may cause a shift in substrate oxidation from carbohydrate to fatty acid oxidation after hypoxic exposure, which could influence the acute exercise-induced response of lipids and lipoproteins. (Workman and Basset, 2012; Kelly and Basset, 2017; Mahat et al., 2018). Unfortunately, there is currently no adequate explanation for the acute effects of IHHE on LDL-C concentration observed in the present study. Thus, further studies are needed to verify the results of the present study and to unravel the potential underlying mechanisms.

The lipid and lipoprotein profile, especially LDL-C and Tgl blood concentrations, is an important index for the assessment of the overall risk for atherosclerotic cardiovascular disease at any age (Grundy et al., 2019). However, the results of the present study indicate that an IHHE program applied prior to aerobic cycling exercise did not promote additional effects compared to the same exercise program without IHHE on basal blood lipid and lipoprotein concentrations in geriatric patients. This is consistent with a previous study examining the effect of adding an IHHE program to a multimodal training intervention (5–7 weeks, 2-3 sessions week^−1^) in geriatric patients (Bayer et al., 2017a). The authors found no differences in blood concentrations of tCh, HDL-C, LDL-C, and Tgl between patients who completed an IHHE program (30–40 min, F_i_O_2_ = 0.10–0.14 for 4–7 min, F_i_O_2_ = 0.30–0.40 for 2–4 min) in addition to a multimodal training and those who completed a multimodal training only. In contrast, studies in patients with metabolic syndrome (Bestavashvili et al., 2021; Bestavashvili et al., 2022), prediabetes (Serebrovska et al., 2019), and coronary arteria disease (Glazachev et al., 2017) found that IHHE promotes beneficial effects on patients’ lipid and lipoprotein profile. For instance, Bestavashvili et al. showed higher absolute changes in tCh (-0.8 vs. 0.3 mmol/L), LDL-C (-0.8 vs. 0.3 mmol/L), and Tgl (-0.3 vs. 0.1 mmol/L) concentrations after 3 weeks (5 sessions∙week^−1^) of IHHE (40–45 min, F_i_O_2_ = 0.11–0.12 for 4–7 min, F_i_O_2_ = 0.30–0.35 for 2–4 min) compared to a control group (sham IHHE: F_i_O_2_ ∼ 0.21 for 40–45 min) in patients with metabolic syndrome (Bestavashvili et al., 2021). Similar results were found by Bestavashvili et al. in another study for tCh (-0.8 vs. 0.3) and LDL-C levels (-0.8 vs. 0.3) using a similar IHHE program in patients with metabolic syndrome (Bestavashvili et al., 2022). However, in both studies, the baseline lipid and lipoprotein concentrations (i.e., tCh, LDL-C and Tgl concentration) were statistically higher in patients who completed the IHHE program than in those who underwent sham IHHE. Therefore, it might be possible that these differences at the pre-test influenced the results and that these should be interpreted with caution. In addition, Serebrovska et al. demonstrated decreased blood tCh and LDL-C concentrations after 3 weeks (5 sessions∙week^−1^) of IHHE (∼32 min, F_i_O_2_ = 0.12 for 5 min, F_i_O_2_ = 0.33 for 3 min) in patients with prediabetes (Serebrovska et al., 2019). However, the authors found no differences compared to a control group receiving sham IHHE. Another study reported decreased within-group tCh and LDL-C levels as well as a reduced atherogenic index (i.e., [tCh–HDL-C] ÷ HDL-C) after 5 weeks (3 session∙week^−1^) of IHHE (F_i_O_2_ = 0.10–0.12 for 4–6 min, F_i_O_2_ = 0.30–0.35 for 3 min, 5-7 cycles∙session^−1^) in patients with coronary artery disease (Glazachev et al., 2017). Furthermore, the authors have found that tCh and LDL-C blood concentrations as well as the atherogenic index was lower in patients who received the IHHE program than in those patients who completed a standard rehabilitation program lasting 8 weeks (16 sessions in total). Nevertheless, it should be noted that the patients who underwent the standard rehabilitation program were enrolled after its completion and their baseline values (i.e., before completing the standard rehabilitation program) were not considered in the analysis and interpretation of the results. Hence, there is currently no robust evidence supporting a beneficial effect of IHHE on blood lipid and lipoprotein profile, at least over a duration of 3–7 weeks. In this regard, a systematic review suggested that interventions using active hypoxic methods (e.g., continuous hypoxic training) require an intervention duration of more than 4 and up to 8 weeks to elicit further improvements in blood lipid and lipoprotein concentrations (Hobbins et al., 2017). In theory, hypoxia could induce positive effects on body weight and composition potentially due to increased energy expenditure and lipid metabolism (Workman and Basset, 2012) and/or decreased energy intake mediated by the regulation of appetite mechanisms (Bailey et al., 2015; Debevec, 2017). Over a longer duration, this could lead to improvements in metabolic markers and a reduction in overall risk for atherosclerotic cardiovascular disease (Poobalan et al., 2004). Importantly, when intermittent hypoxia is applied over a long duration, it has been suggested that the hypoxic dose must be progressively adjusted throughout the intervention period to avoid a possible plateau in adaptations such as body composition (Gatterer et al., 2015; Hobbins et al., 2017). To date, however, such a protocol has not been performed using IHHE.

Beside blood lipid and lipoprotein concentrations, blood pressure also represents an important modifiable risk factor for cardiovascular disease with an extensive global impact (Yusuf et al., 2020). There is rather solid evidence supporting the assumption that hypoxic exposure at rest (e.g., intermittent hypoxic exposure) or in combination with exercise (e.g., aerobic exercise [continuous or intermittent hypoxic training]) elicits beneficial hypotensive effects (Verges et al., 2015; Wee and Climstein, 2015) and improves vascular health (Montero and Lundby, 2016). A previous review reported decreases of 10–30 mmHg and 10–15 mmHg in SBP and DBP, respectively, in patients with type I to II hypertension after intermittent or prolonged hypoxic exposure (Serebrovskaya et al., 2008). Considering that every 10 mmHg reduction in SBP or 5 mmHg reduction in DBP is associated with a 20% reduction in the risk of major cardiovascular events (Ettehad et al., 2016), the hypotensive effects of well-dosed hypoxic interventions are practically relevant for the prevention and treatment of cardiovascular disease. This is in accordance with the results of the present study, indicating that the addition of IHHE prior to aerobic cycling exercise induced a more pronounced decrease in SBP than aerobic cycling alone (8.9 mmHg vs. 1.4 mmHg). However, a significant reduction in SBP could only be detected between the 4th week (4-weeks mid-training) and the end (post-training) of the intervention. This could be explained by a relatively high standard deviation in pre-training values as well as a delayed onset of hypotensive effects due to the IHHE (Verges et al., 2015; Montero and Lundby, 2016). In addition, no effect on DBP was observed, which could be further explained by the fact that DBP averaged < 80 mmHg in both groups at baseline. Due to the higher reduction of the SBP in patients who performed the IHHE program prior to aerobic cycling, it could be assumed that the additional exposure to intermittent hypoxia and hyperoxia elicited further mechanisms associated with hypotensive effects such as increased vascularisation (Semenza, 2014) and/or vasodilatation (Muangritdech et al., 2020) and/or reduced sympathetic activation (Serebrovskaya et al., 2008; Lizamore et al., 2016). In contrast, Bayer et al. reported no improvement in SBP among geriatric patients after a 5–7 weeks IHHE program conducted at 2–3 days per week in addition to a multimodal training intervention (Bayer et al., 2017b). However, the authors did not include multiple time points to assess the short-term effect on blood pressure.

In addition, it should be considered that most of the patients participating in the present study have taken hypotensive drugs (e.g., β-blockers, ACE inhibitors, calcium channel blockers, diuretics), which could have led to an interaction with the aerobic training and/or IHHE (Kendrick et al., 1987). However, we did not observe any acute side effects. With regard to studies using intermittent hypoxia-normoxia, Burtscher et al. exposed normally physically active males with NYHA class I and II to 3 weeks of intermittent hypoxia (3–5 min of hypoxia [F_i_O_2_ = 0.10–0.14] interspersed by 3 min of normoxia, 3-5 cycles∙session^−1^, 5 sessions∙week^−1^) and found a decrease of 5.5% (∼9.0 mmHg) in SBP during submaximal exercise (i.e., cycling at a load corresponding to 1 W/kg body weight) (Burtscher et al., 2004). Furthermore, Lyamina et al. reported that the average SBP and DBP (i.e., obtained by 24-h monitoring) decreased by 15% (∼22.0 mmHg) and 17% (∼16.6 mmHg), respectively, after 20 consecutive days of intermittent hypoxic exposure (3 min of hypoxia [F_i_O_2_ = 0.10] interspersed by 3 min of normoxia, 4–10 cycles∙session^−1^) in young males with stage I hypertension (Lyamina et al., 2011). The greater hypotensive effects observed by Lyamina et al. compared to our findings could be explained by the higher inter-session frequency (i.e., 3 vs. 7 sessions∙week^−1^) and/or the differences in patients’ characteristics (e.g., geriatric patients vs. young males with stage I hypertension).

Although, it has been hypothesized that replacing normoxia with hyperoxia can increase the adaptive response to the intermittent hypoxic stimulus by upregulating reactive oxygen species and hypoxia-inducible genes (Sazontova et al., 2012; Mallet et al., 2020), there is currently no study available that has directly examined the effects of intermittent exposure to hypoxia-normoxia and hypoxia-hyperoxia on blood pressure and its underlying mechanisms (e.g., NO generation) in humans. In this regard, Serebrovska et al. investigated the effect of 3 weeks (5 sessions∙week^−1^) of IHHE (5 min of hypoxia [F_i_O_2_ = 0.12] interspersed by 3 min of hyperoxia [F_i_O_2_ = 0.33], 4 cycles∙session^−1^), intermittent hypoxic exposure (5 min of hypoxia [F_i_O_2_ = 0.12] interspersed by 5 min of normoxia, 4 cycles∙session^−1^), and sham hypoxia on carbohydrate and lipid metabolism in prediabetic patients (Serebrovska et al., 2019). The authors observed that both hypoxia protocols were similarly effective in reducing fasting and 2 h post oral glucose tolerance test glucose level as well as decreasing tCh and LDL-C blood concentration. The authors speculated that an advantage of IHHE over intermittent hypoxic exposure may be a faster reoxygenation resulting in a shorter session duration (Serebrovska et al., 2019). However, further studies are required to directly compare the effects of IHHE and intermittent hypoxic exposure on, for example, changes in cardiometabolic risk factors, physical performance, cognitive performance as well as changes at the molecular and cellular level (Behrendt et al., 2022b).

## 5 Limitations

The present study has a few limitations that need to be considered. First, the experimental design did not include a group that performed IHHE only to analyze its independent acute and chronic effects on blood lipid and lipoprotein concentration as well as resting blood pressure. Second, because nutrition intake may affect blood lipid and lipoprotein concentrations, it should be noted that patients’ diets were not monitored. Even though all patients received standardized meals within their facilities, some dietary habits may have changed during the duration of the intervention period. Third, blood samples were not analyzed regarding the potential changes in plasma volume. Acute exercise-induced changes in plasma volume are known to affect serum or plasma lipid and lipoprotein concentrations (Pronk, 1993). Therefore, it is recommended to correct the levels of lipids and lipoproteins whenever significant changes in plasma volume have affected the results (Kargotich et al., 1998). Although, a previous study showed that plasma volume did not change after 1 h and 2 h of low-intensity aerobic exercise (walking at a velocity equivalent to 30% of individuals’ maximal oxygen uptake) in trained and untrained young adults (Pay et al., 1992), it cannot be assumed with confidence that plasma volume did not change in the present study. Fourth, we did not investigate mechanisms that might underline the antihypertensive effect of IHHE, such as NO synthase.

## 6 Conclusion

The present study provides first hints that IHHE prior to aerobic cycling can influence the acute exercise-related responses in LDL-C concentration in geriatric patients. Furthermore, the present results suggest that 17 training sessions of IHHE prior to aerobic cycling or sole aerobic cycling did not induce changes in basal lipid or lipoprotein concentrations. Further investigations are warranted to identify the long-term effects of IHHE on lipid and lipoprotein concentrations as well as its synergistic effect with exercise or training. Beside blood lipids and lipoproteins, the current study showed that an additional IHHE prior to aerobic cycling seems to be more effective to reduce SBP in geriatric patients in comparison to aerobic cycling alone after 6 weeks of training. Based on the results obtained, individually tailored IHHE prior to aerobic cycling is well tolerated by geriatric patients and effective in eliciting hypotensive effects. Therefore, IHHE might be a promising non-pharmacological intervention strategy to reduce cardiovascular risk factors in vulnerable populations.

## Data Availability

The raw data supporting the conclusions of this article will be made available by the authors, without undue reservation.

## References

[B1] Alshami A. Romero C. Avila A. Varon J. (2018). Management of hypertensive crises in the elderly. J. Geriatr. Cardiol. 15, 504–512. 10.11909/j.issn.1671-5411.2018.07.007 30364798 PMC6198269

[B2] Alvis B. D. Hughes C. G. (2015). Physiology considerations in geriatric patients. Anesthesiol. Clin. 33, 447–456. 10.1016/j.anclin.2015.05.003 26315630 PMC4556136

[B3] Antunes B. M. Rossi F. E. Oyama L. M. Rosa-Neto J. C. Lira F. S. (2020). Exercise intensity and physical fitness modulate lipoproteins profile during acute aerobic exercise session. Sci. Rep. 10, 4160. 10.1038/s41598-020-61039-6 32139762 PMC7058045

[B4] Cholesterol Treatment Trialsists' Collaboration, Baigent C. Blackwell L. Emberson J. Holland L. E. Reith C. Bhala N. (2010). Efficacy and safety of more intensive lowering of LDL cholesterol: A meta-analysis of data from 170 000 participants in 26 randomised trials. Lancet 376, 1670–1681. 10.1016/S0140-6736(10)61350-5 21067804 PMC2988224

[B5] Bailey D. P. Smith L. R. Chrismas B. C. Taylor L. Stensel D. J. Deighton K. (2015). Appetite and gut hormone responses to moderate-intensity continuous exercise versus high-intensity interval exercise, in normoxic and hypoxic conditions. Appetite 89, 237–245. 10.1016/j.appet.2015.02.019 25700630

[B6] Barrio-Cortes J. Castaño-Reguillo A. Beca-Martínez M. T. Bandeira-de Oliveira M. López-Rodríguez C. Jaime-Sisó M. Á. (2021). Chronic diseases in the geriatric population: Morbidity and use of primary care services according to risk level. BMC Geriatr. 21, 278. 10.1186/s12877-021-02217-7 33902470 PMC8074273

[B7] Bayer U. Glazachev O. S. Likar R. Burtscher M. Kofler W. Pinter G. (2017a). Adaptation to intermittent hypoxia-hyperoxia improves cognitive performance and exercise tolerance in the elderly. Adv. Gerontol. 7, 214–220. 10.1134/S2079057017030031 28575566

[B8] Bayer U. Likar R. Pinter G. Stettner H. Demschar S. Trummer B. (2017b). Intermittent hypoxic-hyperoxic training on cognitive performance in geriatric patients. Alzheimers Dement. 3, 114–122. 10.1016/j.trci.2017.01.002 PMC5651371 29067323

[B9] Behrendt T. Bielitzki R. Behrens M. Glazachev O. S. Schega L. (2022a). Effects of intermittent hypoxia-hyperoxia exposure prior to aerobic cycling exercise on physical and cognitive performance in geriatric patients—a randomized controlled trial. Front. Physiol. 13, 899096. 10.3389/fphys.2022.899096 35694402 PMC9178199

[B10] Behrendt T. Bielitzki R. Behrens M. Herold F. Schega L. (2022b). Effects of intermittent hypoxia-hyperoxia on performance- and health-related outcomes in humans: A systematic review. Sports Med. Open 8, 70. 10.1186/s40798-022-00450-x 35639211 PMC9156652

[B11] Bestavashvili A. A. Glazachev O. S. Bestavashvili A. A. Ines D. Suvorov A. Y. Vorontsov N. V. (2021). The effects of intermittent hypoxic–hyperoxic exposures on lipid profile and inflammation in patients with metabolic syndrome. Front. Cardiovasc. Med. 8, 700826. 10.3389/fcvm.2021.700826 34513946 PMC8429814

[B12] Bestavashvili A. Glazachev O. Bestavashvili A. Suvorov A. Zhang Y. Zhang X. (2022). Intermittent hypoxic-hyperoxic exposures effects in patients with metabolic syndrome: Correction of cardiovascular and metabolic profile. Biomedicines 10, 566. 10.3390/biomedicines10030566 35327372 PMC8945352

[B13] Blanca M. J. Alarcón R. Arnau J. Bono R. Bendayan R. (2017). Non-normal data: Is ANOVA still a valid option? Psicothema 29, 552–557. 10.7334/psicothema2016.383 29048317

[B14] Borg G. A. (1982). Psychophysical bases of perceived exertion. Med. Sci. Sports Exerc. 14, 377–381. 10.1249/00005768-198205000-00012 7154893

[B15] Bounds R. G. Grandjean P. W. O'Brien B. C. Inman C. Crouse S. F. (2000). Diet and short term plasma lipoprotein-lipid changes after exercise in trained Men. Int. J. Sport Nutr. Exerc. Metab. 10, 114–127. 10.1123/ijsnem.10.2.114 10861333

[B16] Burtscher J. Mallet R. T. Pialoux V. Millet G. P. Burtscher M. (2022). Adaptive responses to hypoxia and/or hyperoxia in humans. Antioxid. Redox Signal. [Epub ahead of print]. 10.1089/ars.2021.0280 35102747

[B17] Burtscher M. Pachinger O. Ehrenbourg I. Mitterbauer G. Faulhaber M. Pühringer R. (2004). Intermittent hypoxia increases exercise tolerance in elderly men with and without coronary artery disease. Int. J. Cardiol. 96, 247–254. 10.1016/j.ijcard.2003.07.021 15262041

[B18] Cohen J. (1992). Quantitative methods in psychology: A power primer. Psychol. Bull. 112, 155–159. 10.1037//0033-2909.112.1.155 19565683

[B19] Cohen J. (2013). Statistical power analysis for the behavioral Sciences. New York, United States: Routledge.

[B20] Cornelissen V. A. Smart N. A. (2013). Exercise training for blood pressure: A systematic review and meta-analysis. J. Am. Heart Assoc. 2, e004473. 10.1161/JAHA.112.004473 23525435 PMC3603230

[B21] Costalat G. Lemaitre F. Tobin B. Renshaw G. (2018). Intermittent hypoxia revisited: A promising non-pharmaceutical strategy to reduce cardio-metabolic risk factors? Sleep. Breath. 22, 267–271. 10.1007/s11325-017-1459-8 28155101

[B22] Debevec T. (2017). Hypoxia-related hormonal appetite modulation in humans during rest and exercise: Mini review. Front. Physiol. 8, 366. 10.3389/fphys.2017.00366 28611686 PMC5447736

[B23] Dudnik E. Zagaynaya E. Glazachev O. S. Susta D. (2018). Intermittent hypoxia-hyperoxia conditioning improves cardiorespiratory fitness in older comorbid cardiac outpatients without hematological changes: A randomized controlled trial. High. Alt. Med. Biol. 19, 339–343. 10.1089/ham.2018.0014 30251879

[B24] Duennwald T. Gatterer H. Groop P.-H. Burtscher M. Bernardi L. (2013). Effects of a single bout of interval hypoxia on cardiorespiratory control and blood glucose in patients with type 2 diabetes. Diabetes Care 36, 2183–2189. 10.2337/dc12-2113 23536585 PMC3714488

[B25] Ericsson S. Eriksson M. Vitols S. Einarsson K. Berglund L. Angelin B. (1991). Influence of age on the metabolism of plasma low density lipoproteins in healthy males. J. Clin. Invest. 87, 591–596. 10.1172/JCI115034 1991842 PMC296347

[B26] Ettehad D. Emdin C. A. Kiran A. Anderson S. G. Callender T. Emberson J. (2016). Blood pressure lowering for prevention of cardiovascular disease and death: A systematic review and meta-analysis. Lancet 387, 957–967. 10.1016/S0140-6736(15)01225-8 26724178

[B27] Ferguson M. A. Alderson N. L. Trost S. G. Essig D. A. Burke J. R. Durstine J. L. (1998). Effects of four different single exercise sessions on lipids, lipoproteins, and lipoprotein lipase. J. Appl. Physiol. 85, 1169–1174. 10.1152/jappl.1998.85.3.1169 9729596

[B28] Fittipaldi E. O. d. S. Dornelas de Andrade A. Santos A. C. O. Campos S. L. Souza H. C. M. d. Fernandes J. (2020). Cardiorespiratory performance and acute effect of high-intensity exercise on lipid profile in hypertensive sedentary older adults with and without diabetes mellitus. Arch. Gerontol. Geriatr. 89, 104061. 10.1016/j.archger.2020.104061 32325307

[B29] Folstein M. F. Folstein S. E. McHugh P. R. (1975). “Mini-mental state”. A practical method for grading the cognitive state of patients for the clinician J. Psychiatr. Res. 12, 189–198. 10.1016/0022-3956(75)90026-6 1202204

[B30] Forsythe J. A. Jiang B. H. Iyer N. V. Agani F. Leung S. W. Koos R. D. (1996). Activation of vascular endothelial growth factor gene transcription by hypoxia-inducible factor 1. Mol. Cell. Biol. 16, 4604–4613. 10.1128/MCB.16.9.4604 8756616 PMC231459

[B31] Gatterer H. Haacke S. Burtscher M. Faulhaber M. Melmer A. Ebenbichler C. (2015). Normobaric intermittent hypoxia over 8 Months does not reduce body weight and metabolic risk factors-a randomized, single blind, placebo-controlled study in normobaric hypoxia and normobaric sham hypoxia. Obes. Facts 8, 200–209. 10.1159/000431157 26008855 PMC5644878

[B32] GBD 2017 Risk Factor Collaborators (2018). Global, regional, and national comparative risk assessment of 84 behavioural, environmental and occupational, and metabolic risks or clusters of risks for 195 countries and territories, 1990–2017: A systematic analysis for the global burden of disease study 2017. Lancet 392, 1923–1994. 10.1016/S0140-6736(18)32225-6 30496105 PMC6227755

[B33] Glazachev O. S. Kopylov P. Susta D. Dudnik E. Zagaynaya E. (2017). Adaptations following an intermittent hypoxia-hyperoxia training in coronary artery disease patients: A controlled study. Clin. Cardiol. 40, 370–376. 10.1002/clc.22670 28323322 PMC6490434

[B34] Greene N. P. Martin S. E. Crouse S. F. (2012). Acute exercise and training alter blood lipid and lipoprotein profiles differently in overweight and obese men and women. Obes. (Silver Spring) 20, 1618–1627. 10.1038/oby.2012.65 22421926

[B35] Griep M. I. Borg E. Collys K. Massart D. L. (1998). Category ratio scale as an alternative to magnitude matching for age-related taste and odour perception. Food Qual. Prefer. 9, 67–72. 10.1016/S0950-3293(97)00030-X

[B36] Grundy S. M. Stone N. J. Bailey A. L. Beam C. Birtcher K. K. Blumenthal R. S. (2019). 2018 AHA/ACC/AACVPR/AAPA/ABC/ACPM/ADA/AGS/APhA/ASPC/NLA/PCNA guideline on the management of blood cholesterol: Executive summary: A report of the American college of cardiology/American heart association Task Force on Clinical practice guidelines. J. Am. Coll. Cardiol. 73, 3168–3209. 10.1016/j.jacc.2018.11.002 30423391

[B37] Gude N. A. Broughton K. M. Firouzi F. Sussman M. A. (2018). Cardiac ageing: Extrinsic and intrinsic factors in cellular renewal and senescence. Nat. Rev. Cardiol. 15, 523–542. 10.1038/s41569-018-0061-5 30054574

[B38] Hairi N. N. Cumming R. G. Naganathan V. Handelsman D. J. Le Couteur D. G. Creasey H. (2010). Loss of muscle strength, mass (sarcopenia), and quality (specific force) and its relationship with functional limitation and physical disability: The concord health and ageing in men project. J. Am. Geriatr. Soc. 58, 2055–2062. 10.1111/j.1532-5415.2010.03145.x 21054284

[B39] Hannan A. L. Hing W. Simas V. Climstein M. Coombes J. S. Jayasinghe R. (2018). High-intensity interval training versus moderate-intensity continuous training within cardiac rehabilitation: A systematic review and meta-analysis. Open Access J. Sports Med. 9, 1–17. 10.2147/OAJSM.S150596 29416382 PMC5790162

[B40] Havlicek L. L. Peterson N. L. (1974). Robustness of the T test: A guide for researchers on effect of violations of assumptions. Psychol. Rep. 34, 1095–1114. 10.2466/pr0.1974.34.3c.1095

[B41] Hayes H. B. Jayaraman A. Herrmann M. Mitchell G. S. Rymer W. Z. Trumbower R. D. (2014). Daily intermittent hypoxia enhances walking after chronic spinal cord injury: A randomized trial. Neurology 82, 104–113. 10.1212/01.WNL.0000437416.34298.43 24285617 PMC3897437

[B42] Hedayatnia M. Asadi Z. Zare-Feyzabadi R. Yaghooti-Khorasani M. Ghazizadeh H. Ghaffarian-Zirak R. (2020). Dyslipidemia and cardiovascular disease risk among the MASHAD study population. Lipids Health Dis. 19, 42. 10.1186/s12944-020-01204-y 32178672 PMC7075010

[B43] Herrera M. D. Mingorance C. Rodríguez-Rodríguez R. Alvarez de Sotomayor M. (2010). Endothelial dysfunction and aging: An update. Ageing Res. Rev. 9, 142–152. 10.1016/j.arr.2009.07.002 19619671

[B44] Hicks A. L. MacDougall J. D. Muckle T. J. (1987). Acute changes in high-density lipoprotein cholesterol with exercise of different intensities. J. Appl. Physiol. 63, 1956–1960. 10.1152/jappl.1987.63.5.1956 3693229

[B45] Hilmer S. N. McLachlan A. J. Le Couteur D. G. (2007). Clinical pharmacology in the geriatric patient. Fundam. Clin. Pharmacol. 21, 217–230. 10.1111/j.1472-8206.2007.00473.x 17521291

[B46] Hobbins L. Hunter S. Gaoua N. Girard O. (2017). Normobaric hypoxic conditioning to maximize weight loss and ameliorate cardio-metabolic health in obese populations: A systematic review. Am. J. Physiol. Regul. Integr. Comp. Physiol. 313, R251–R264. 10.1152/ajpregu.00160.2017 28679682

[B47] Hodson L. Skeaff C. M. Fielding B. A. (2008). Fatty acid composition of adipose tissue and blood in humans and its use as a biomarker of dietary intake. Prog. Lipid Res. 47, 348–380. 10.1016/j.plipres.2008.03.003 18435934

[B48] Holthoff V. A. Marschner K. Scharf M. Steding J. Meyer S. Koch R. (2015). Effects of physical activity training in patients with alzheimer's dementia: Results of a pilot RCT study. PLoS ONE 10, e0121478. 10.1371/journal.pone.0121478 25884637 PMC4401690

[B49] Hoogendam Y. Y. Hofman A. van der Geest J. N. van der Lugt A. Ikram M. A. (2014). Patterns of cognitive function in aging: The rotterdam study. Eur. J. Epidemiol. 29, 133–140. 10.1007/s10654-014-9885-4 24553905

[B50] Horscroft J. A. Murray A. J. (2014). Skeletal muscle energy metabolism in environmental hypoxia: Climbing towards consensus Extrem. Physiol. Med. 19, 19–17. 10.1186/2046-7648-3-19 PMC4253994 25473486

[B51] Hu R. Dai A. Tan S. (2002). Hypoxia-inducible factor 1 alpha upregulates the expression of inducible nitric oxide synthase gene in pulmonary arteries of hyposic rat. Chin. Med. J. 115, 1833–1837. 12622934

[B52] Hughes R. A. Housh T. J. Hughes R. J. Johnson G. O. (1991). The effect of exercise duration on serum cholesterol and triglycerides in women. Res. Q. Exerc. Sport 62, 98–104. 10.1080/02701367.1991.10607525 2028100

[B53] Imamura H. Katagiri S. Uchida K. Miyamoto N. Nakano H. Shirota T. (2000). Acute effects of moderate exercise on serum lipids, lipoproteins and apolipoproteins in sedentary young women. Clin. Exp. Pharmacol. Physiol. 27, 975–979. 10.1046/j.1440-1681.2000.03384.x 11117233

[B54] IzquierdoM.MerchantR. A.MorleyJ. E.AnkerS. D.AprahamianI.AraiH. (2021). International exercise recommendations in older adults (ICFSR): Expert consensus guidelines. J. Nutr. Health Aging 25, 824–853. 10.1007/s12603-021-1665-8 34409961

[B55] Jun J. C. Shin M.-K. Devera R. Yao Q. Mesarwi O. Bevans-Fonti S. (2014). Intermittent hypoxia-induced glucose intolerance is abolished by α-adrenergic blockade or adrenal medullectomy. Am. J. Physiol. Endocrinol. Metab. 307, E1073–E1083. 10.1152/ajpendo.00373.2014 25315697 PMC4254988

[B56] Kalpouzos G. Persson J. Nyberg L. (2012). Local brain atrophy accounts for functional activity differences in normal aging. Neurobiol. Aging 33, e1–e1623. e13. 10.1016/j.neurobiolaging.2011.02.021 21524432

[B57] Kargotich S. Goodman C. Keast D. Morton A. R. (1998). The influence of exercise-induced plasma volume changes on the interpretation of biochemical parameters used for monitoring exercise, training and sport. Sports Med. 26, 101–117. 10.2165/00007256-199826020-00004 9777683

[B112] Kendrick Z. V. Cristal N. Lowenthal D. T. (1987). Cardiovascular drugs and exercise interactions. Cardiol. Clin. 5, 227–44. 10.1016/s0733-8651(18)30548-4 3555797

[B58] Keller K. Engelhardt M. (2013). Strength and muscle mass loss with aging process. Age and strength loss. Muscle Ligaments Tendons J. 3, 346–350. 10.32098/mltj.04.2013.17 PMC3940510 24596700

[B59] Kelly L. P. Basset F. A. (2017). Acute normobaric hypoxia increases post-exercise lipid oxidation in healthy males. Front. Physiol. 8, 293. 10.3389/fphys.2017.00293 28567018 PMC5434119

[B60] Lakens D. (2013). Calculating and reporting effect sizes to facilitate cumulative science: A practical primer for t-tests and ANOVAs. Front. Psychol. 4, 863. 10.3389/fpsyg.2013.00863 24324449 PMC3840331

[B61] Lionakis N. Mendrinos D. Sanidas E. Favatas G. Georgopoulou M. (2012). Hypertension in the elderly. World J. Cardiol. 4, 135–147. 10.4330/wjc.v4.i5.135 22655162 PMC3364500

[B62] Lizamore C. A. Hamlin M. J. (2017). The use of simulated altitude techniques for beneficial cardiovascular health outcomes in nonathletic, sedentary, and clinical populations: A literature review. High. Alt. Med. Biol. 18, 305–321. 10.1089/ham.2017.0050 28846046

[B63] Lizamore C. A. Kathiravel Y. Elliott J. Hellemans J. Hamlin M. J. (2016). The effect of short-term intermittent hypoxic exposure on heart rate variability in a sedentary population. Physiol. Int. 103, 75–85. 10.1556/036.103.2016.1.7 27030629

[B64] Louis M. Punjabi N. M. (2009). Effects of acute intermittent hypoxia on glucose metabolism in awake healthy volunteers. J. Appl. Physiol. 106, 1538–1544. 10.1152/japplphysiol.91523.2008 19265062 PMC2681331

[B65] Lyamina N. P. Lyamina S. V. Senchiknin V. N. Mallet R. T. Downey H. F. Manukhina E. B. (2011). Normobaric hypoxia conditioning reduces blood pressure and normalizes nitric oxide synthesis in patients with arterial hypertension. J. Hypertens. 29, 2265–2272. 10.1097/HJH.0b013e32834b5846 21897291

[B66] Mahat B. Chassé É. Lindon C. Mauger J.-F. Imbeault P. (2018). No effect of acute normobaric hypoxia on plasma triglyceride levels in fasting healthy men. Appl. Physiol. Nutr. Metab. 43, 727–732. 10.1139/apnm-2017-0505 29466682

[B67] Mallet R. T. Burtscher J. Manukhina E. B. Downey H. F. Glazachev O. S. Serebrovskaya T. V. (2020). “Hypoxic–hyperoxic conditioning and dementia,” in Diagnosis and management in dementia (Elsevier), 745–760.

[B68] Mancia G. Fagard R. Narkiewicz K. Redon J. Zanchetti A. Böhm M. (2013). 2013 ESH/ESC guidelines for the management of arterial hypertension: The task force for the management of arterial hypertension of the European society of hypertension (ESH) and of the European society of cardiology (ESC). Eur. Heart J. 34, 2159–2219. 10.1093/eurheartj/eht151 23771844

[B69] Masnoon N. Shakib S. Kalisch-Ellett L. Caughey G. E. (2017). What is polypharmacy? A systematic review of definitions. BMC Geriatr. 17, 230. 10.1186/s12877-017-0621-2 29017448 PMC5635569

[B70] Mau-Moeller A. Behrens M. Lindner T. Bader R. Bruhn S. (2013). Age-related changes in neuromuscular function of the quadriceps muscle in physically active adults. J. Electromyogr. Kinesiol. 23, 640–648. 10.1016/j.jelekin.2013.01.009 23453325

[B71] Montero D. Lundby C. (2016). Effects of exercise training in hypoxia versus normoxia on vascular health. Sports Med. 46, 1725–1736. 10.1007/s40279-016-0570-5 27286988

[B72] Muangritdech N. Hamlin M. J. Sawanyawisuth K. Prajumwongs P. Saengjan W. Wonnabussapawich P. (2020). Hypoxic training improves blood pressure, nitric oxide and hypoxia-inducible factor-1 alpha in hypertensive patients. Eur. J. Appl. Physiol. 120, 1815–1826. 10.1007/s00421-020-04410-9 32524226

[B73] Newhouse L. P. Joyner M. J. Curry T. B. Laurenti M. C. Man C. D. Cobelli C. (2017). Three hours of intermittent hypoxia increases circulating glucose levels in healthy adults. Physiol. Rep. 5, e13106. 10.14814/phy2.13106 28087818 PMC5256164

[B74] Okada Y. Galbreath M. M. Shibata S. Jarvis S. S. VanGundy T. B. Meier R. L. (2012). Relationship between sympathetic baroreflex sensitivity and arterial stiffness in elderly men and women. Hypertension 59, 98–104. 10.1161/HYPERTENSIONAHA.111.176560 22106403 PMC3241909

[B75] Pagano R. R. (2009). Understanding statistics in the behavioral Sciences. Belmont: Wadsworth Cengage Learning.

[B76] Pareja-Galeano H. Garatachea N. Lucia A. (2015). Exercise as a polypill for chronic diseases. Prog. Mol. Biol. Transl. Sci. 135, 497–526. 10.1016/bs.pmbts.2015.07.019 26477928

[B77] Pay H. E. Hardman A. E. Jones G. J. W. Hudson A. (1992). The acute effects of low-intensity exercise on plasma lipids in endurance-trained and untrained young adults. Eur. J. Appl. Physiol. Occup. Physiol. 64, 182–186. 10.1007/BF00717958 1555566

[B78] Pazan F. Wehling M. (2021). Polypharmacy in older adults: A narrative review of definitions, epidemiology and consequences. Eur. Geriatr. Med. 12, 443–452. 10.1007/s41999-021-00479-3 33694123 PMC8149355

[B79] Pescatello L. S. MacDonald H. V. Lamberti L. Johnson B. T. (2015). Exercise for hypertension: A prescription update integrating existing recommendations with emerging research. Curr. Hypertens. Rep. 17, 87. 10.1007/s11906-015-0600-y 26423529 PMC4589552

[B80] Pickering T. G. Hall J. E. Appel L. J. Falkner B. E. Graves J. Hill M. N. (2005). Recommendations for blood pressure measurement in humans and experimental animals: Part 1: Blood pressure measurement in humans: A statement for professionals from the subcommittee of professional and public education of the American heart association council on high blood pressure research. Hypertension 45, 142–161. 10.1161/01.HYP.0000150859.47929.8e 15611362

[B81] Poobalan A. Aucott L. Smith W. C. S. Avenell A. Jung R. Broom J. (2004). Effects of weight loss in overweight/obese individuals and long-term lipid outcomes-a systematic review. Obes. Rev. 5, 43–50. 10.1111/j.1467-789X.2004.00127.x 14969506

[B82] Pramsohler S. Burtscher M. Faulhaber M. Gatterer H. Rausch L. Eliasson A. (2017). Endurance training in normobaric hypoxia imposes less physical stress for geriatric rehabilitation. Front. Physiol. 8, 514. 10.3389/fphys.2017.00514 28785224 PMC5517449

[B83] Pronk N. P. (1993). Short term effects of exercise on plasma lipids and lipoproteins in humans. Sports Med. 16, 431–448. 10.2165/00007256-199316060-00006 8303142

[B84] Rafacho A. Gonçalves-Neto L. M. Ferreira F. B. D. Protzek A. O. P. Boschero A. C. Nunes E. A. (2013). Glucose homoeostasis in rats exposed to acute intermittent hypoxia. Acta Physiol. 209, 77–89. 10.1111/apha.12118 23692825

[B85] Reuter E.-M. Behrens M. Zschorlich V. R. (2015). Age-related differences in corticomotor facilitation indicate dedifferentiation in motor planning. Exp. Gerontol. 65, 79–84. 10.1016/j.exger.2015.03.008 25794937

[B86] Robinson M. M. Dasari S. Konopka A. R. Johnson M. L. Manjunatha S. Esponda R. R. (2017). Enhanced protein translation underlies improved metabolic and physical adaptations to different exercise training modes in young and old humans. Cell Metab. 25, 581–592. 10.1016/j.cmet.2017.02.009 28273480 PMC5423095

[B87] Sazontova T. G. Bolotova A. V. Bedareva I. V. Kostina N. V. Arkhipenko Y. V. (2012). “Adaptation to intermittent hypoxia/hyperoxia enhances efficiency of exercise training,” in Intermittent hypoxia and human diseases. Editors Xi L. Serebrovskaya T. V. (London: Springer London), 191–205.

[B88] Scarpulla R. C. (2011). Metabolic control of mitochondrial biogenesis through the PGC-1 family regulatory network. Biochim. Biophys. Acta 1813, 1269–1278. 10.1016/j.bbamcr.2010.09.019 20933024 PMC3035754

[B89] Schega L. Peter B. Törpel A. Mutschler H. Isermann B. Hamacher D. (2013). Effects of intermittent hypoxia on cognitive performance and quality of life in elderly adults: A pilot study. Gerontology 59, 316–323. 10.1159/000350927 23652274

[B90] Schmider E. Ziegler M. Danay E. Beyer L. Bühner M. (2010). Is it really robust? Methodology 6, 147–151. 10.1027/1614-2241/a000016

[B91] Seals D. R. Esler M. D. (2000). Human ageing and the sympathoadrenal system. J. Physiol. 528, 407–417. 10.1111/j.1469-7793.2000.00407.x 11060120 PMC2270159

[B92] Semenza G. L. (2014). Oxygen sensing, hypoxia-inducible factors, and disease pathophysiology. Annu. Rev. Pathol. 9, 47–71. 10.1146/annurev-pathol-012513-104720 23937437

[B93] Serebrovska T. V. Grib O. N. Portnichenko V. I. Serebrovska Z. O. Egorov E. Shatylo V. B. (2019). Intermittent hypoxia/hyperoxia versus intermittent hypoxia/normoxia: Comparative study in prediabetes. High. Alt. Med. Biol. 20, 383–391. 10.1089/ham.2019.0053 31589074

[B94] Serebrovska Z. O. Xi L. Tumanovska L. V. Shysh A. M. Goncharov S. V. Khetsuriani M. (2022). Response of circulating inflammatory markers to intermittent hypoxia-hyperoxia training in healthy elderly people and patients with mild cognitive impairment. Life (Basel) 12, 432. 10.3390/life12030432 35330183 PMC8953753

[B95] Serebrovskaya T. V. Manukhina E. B. Smith M. L. Downey H. F. Mallet R. T. (2008). Intermittent hypoxia: Cause of or therapy for systemic hypertension? Exp. Biol. Med. 233, 627–650. 10.3181/0710-MR-267 18408145

[B96] Serebrovskaya T. V. Xi L. (2016). Intermittent hypoxia training as non-pharmacologic therapy for cardiovascular diseases: Practical analysis on methods and equipment. Exp. Biol. Med. 241, 1708–1723. 10.1177/1535370216657614 PMC4999622 27407098

[B97] Sharrett A. R. Ballantyne C. M. Coady S. A. Heiss G. Sorlie P. D. Catellier D. (2001). Coronary heart disease prediction from lipoprotein cholesterol levels, triglycerides, lipoprotein(a), apolipoproteins A-I and B, and HDL density subfractions: The Atherosclerosis Risk in Communities (ARIC) Study. Circulation 104, 1108–1113. 10.1161/hc3501.095214 11535564

[B98] Stevenson J. C. Crook D. Godsland I. F. (1993). Influence of age and menopause on serum lipids and lipoproteins in healthy women. Atherosclerosis 98, 83–90. 10.1016/0021-9150(93)90225-J 8457253

[B99] The Global Burden of Metabolic Risk Factors for Chronic Diseases Collaboration (2014). Cardiovascular disease, chronic kidney disease, and diabetes mortality burden of cardiometabolic risk factors from 1980 to 2010: A comparative risk assessment, 1687–6121. 10.1016/S2213-8587(14)70102-0 PMC4572741 24842598

[B100] Thompson P. D. Crouse S. F. Goodpaster B. Kelley D. Moyna N. Pescatello L. (2001). The acute versus the chronic response to exercise. Med. Sci. Sports Exerc. 33, S438–S445. 10.1097/00005768-200106001-00012 11427768

[B101] Tuter D. S. Kopylov P. Y. Syrkin A. L. Glazachev O. S. Komarov R. N. Katkov A. I. (2018). Intermittent systemic hypoxic-hyperoxic training for myocardial protection in patients undergoing coronary artery bypass surgery: First results from a single-centre, randomised controlled trial. Open Heart 5, e000891. 10.1136/openhrt-2018-000891 30487981 PMC6241980

[B102] Ungvari Z. Tarantini S. Kiss T. Wren J. D. Giles C. B. Griffin C. T. (2018). Endothelial dysfunction and angiogenesis impairment in the ageing vasculature. Nat. Rev. Cardiol. 15, 555–565. 10.1038/s41569-018-0030-z 29795441 PMC6612360

[B103] van Hulten V. van Meijel R. L. J. Goossens G. H. (2021). The impact of hypoxia exposure on glucose homeostasis in metabolically compromised humans: A systematic review. Rev. Endocr. Metab. Disord. 22, 471–483. 10.1007/s11154-021-09654-0 33851320 PMC8087568

[B104] Vedam H. Phillips C. L. Wang D. Barnes D. J. Hedner J. A. Unger G. (2009). Short-term hypoxia reduces arterial stiffness in healthy men. Eur. J. Appl. Physiol. 105, 19–25. 10.1007/s00421-008-0868-6 18815804

[B105] Verges S. Chacaroun S. Godin-Ribuot D. Baillieul S. (2015). Hypoxic conditioning as a new therapeutic modality. Front. Pediatr. 3, 58. 10.3389/fped.2015.00058 26157787 PMC4476260

[B106] Wahl P. Schmidt A. Demarees M. Achtzehn S. Bloch W. Mester J. (2013). Responses of angiogenic growth factors to exercise, to hypoxia and to exercise under hypoxic conditions. Int. J. Sports Med. 34, 95–100. 10.1055/s-0032-1314815 22918716

[B107] Wee J. Climstein M. (2015). Hypoxic training: Clinical benefits on cardiometabolic risk factors. J. Sci. Med. Sport 18, 56–61. 10.1016/j.jsams.2013.10.247 24268571

[B108] Workman C. Basset F. A. (2012). Post-metabolic response to passive normobaric hypoxic exposure in sedendary overweight males: A pilot study. Nutr. Metab. 9, 103. 10.1186/1743-7075-9-103 PMC3546003 23157699

[B109] Yang H.-C. Lee C.-L. Lin R. Hsu M.-J. Chen C.-H. Lin J.-H. (2014). Effect of biofeedback cycling training on functional recovery and walking ability of lower extremity in patients with stroke. Kaohsiung J. Med. Sci. 30, 35–42. 10.1016/j.kjms.2013.07.006 24388057 PMC11916478

[B110] Yusuf S. Joseph P. Rangarajan S. Islam S. Mente A. Hystad P. (2020). Modifiable risk factors, cardiovascular disease, and mortality in 155 722 individuals from 21 high-income, middle-income, and low-income countries (PURE): A prospective cohort study. Lancet 395, 795–808. 10.1016/S0140-6736(19)32008-2 31492503 PMC8006904

[B111] Zoll J. Ponsot E. Dufour S. Doutreleau S. Ventura-Clapier R. Vogt M. (2006). Exercise training in normobaric hypoxia in endurance runners. III. Muscular adjustments of selected gene transcripts. J. Appl. Physiol. 100, 1258–1266. 10.1152/japplphysiol.00359.2005 16540710

